# Secretion of IFN-γ Associated with Galectin-9 Production by Pleural Fluid Cells from a Patient with Extrapulmonary Tuberculosis

**DOI:** 10.3390/ijms18071382

**Published:** 2017-06-28

**Authors:** Jingge Zhao, Beata Shiratori, Haorile Chagan-Yasutan, Makoto Matsumoto, Toshiro Niki, Michinori Tanaka, Yayoi Takahashi, Osumu Usami, Yugo Ashino, Toshio Hattori

**Affiliations:** 1 Division of Disaster-Related Infectious Diseases, International Research Institute of Disaster Science, Tohoku University, 2-1 Seiryo-machi, Aoba-ku, Sendai, Miyagi 980-8575, Japan; zhaojingge1987@gmail.com (J.Z.); haorile@gmail.com (H.C.-Y.); 2 KKR Tohoku Kosai Hospital, Kokubun-cho 2-3-11, Aoba-ku, Sendai-shi, Miyagi 980-0803, Japan; beatabucekova@gmail.com; 3 Department of Health Science and Social Welfare, Kibi International University, 8 Igamachi, Takahashi 716-8508, Japan; 4 Microbiological Research Institute, Otsuka Pharmaceutical Co., Ltd., Kagasuno, Kawauchi-cho, Tokushima 771-0192, Japan; Matsumoto.Makoto@otsuka.jp; 5 Department of Immunology, Kagawa University, 1-1 Saiwaicho, Takamatsu, Kagawa 760-0016, Japan; niki.t@galpharma.com; 6 Medical Chemistry Research Institute, Otsuka Pharmaceutical Co., Ltd., Kawauchi-cho, Tokushima 771-0192, Japan; Tanaka.Michinori@otsuka.jp; 7 Department of Pathology, Tohoku University Hospital, 1-1 Seiryo-machi, Aoba-ku, Sendai 980-8574, Japan; ytakahashi@patholo2.med.tohoku.ac.jp; 8 Department of Respiratory Medicine, Tohoku University Graduate School of Medicine, 1-1 Seiryo-machi, Aoba-ku, Sendai 980-8574, Japan; usamin@mac.com; 9 Internal Medicine of Respiratory Diseases, Sendai City hospital, Asutonaga-machi, Taihaku-ku, Sendai 982-8502, Japan; ya82@yahoo.co.jp

**Keywords:** tuberculosis, matricellular protein, galectin-9, pleural effusion, ELISPOT

## Abstract

In this study, we investigated the role of a matricellular protein galectin-9 (Gal-9) in pleural effusion related to tuberculosis (TB). Plasma and pleural fluid of a patient with extrapulmonary TB were analyzed for cytokine content by ELISA and Luminex. Peripheral blood mononuclear cells (PBMCs) and pleural fluid cells (PFCs) were examined for interferon-γ (IFN-γ) secretion by the enzyme-linked immunospot (ELISPOT) assay or IFN-γ ELISA, for apoptosis and necrosis by Cell Death Detection ELISA, and also underwent cell sorting. The results indicate that compared to plasma, pleural fluid had increased levels of IFN-γ (1.6 vs. 55.5 pg/mL), IL-10, IL-12p40, vascular endothelial growth factor (VEGF), and Gal-9 (3.0 vs. 936.0 pg/mL), respectively. PFCs culture supernatant exhibited higher concentration of Gal-9 compared to PBMCs in culture, consistent with enriched Gal-9 staining in the granuloma that is in closer vicinity to PFCs compared to PBMCs. PFCS displayed higher IFN-γ secretion after stimulation with TB antigens ESAT-6/CFP-10. Furthermore, in PFCs, Gal-9 alone could stimulate IFN-γ synthesis in culture or ELISPOT, which was inhibited by a Gal-9 antagonist lactose, and which may promote apoptosis and necrosis. These findings suggest that Gal-9 could modulate immune responses and participate in immunopathology of pleural effusion during TB.

## 1. Introduction

Tuberculosis (TB) remains one of the leading causes of mortality worldwide because of its communicability and insufficient diagnostic and treatment measures in low-resource settings [1]. The infection is caused by *Mycobacterium tuberculosis* (MTB) and typically affects the lungs, but can also spread to other parts of the body, in which case it is referred to as extrapulmonary TB. Pleurisy is one of the most common forms of extrapulmonary TB, involving the membranes lining the lungs and chest cavity and resulting in excessive formation of pleural fluid (effusion). Bacterial cultures of pleural fluid reveal MTB less frequently than those of biopsy samples, and sputum or gastric cultures are usually MTB-negative unless pulmonary lesions are present [2]. In TB, pleural fluid contains significantly higher concentrations of interferon-γ (IFN-γ) compared to non-TB pathological conditions [3], which may be a result of T lymphocyte stimulation by MTB antigens [4]. Pleural effusion can be stimulated by various factors. Pleural fluid formation was shown to be associated with vascular endothelial growth factor (VEGF), which induces permeability of the pleural membrane [5], or associated with mast cells recruited by tumors to the pleural space through release of a matricellular protein osteopontin (OPN) in malignant effusion [6]. As mast cells also participate in host defense against MTB infection by regulating secretion of proinflammatory cytokines, which results in the attenuation of granuloma formation [7], they may have a role in pleural effusion during TB.

Galectins are carbohydrate-binding proteins with a variety of functional roles, including danger signaling in innate immunity against infections by acting on several immune cell types [8]. A number of studies indicate a role of galectin-9 (Gal-9) in increased fluid permeability. Thus, Gal-9 was found to be associated with histamine secretion by mast cells in an allergic patient [9] and with dengue virus titers and VEGF secretion in hemorrhagic dengue infection characterized by increasing vascular permeability [10]. Moreover, an association was found between Gal-9 and TB severity, as active TB patients displayed high levels of plasma Gal-9, which correlated with alanine transaminase and creatinine [11]. Gal-9 is a β-galactoside-binding lectin belonging to the family of Matricellular proteins (MCPs) that is expressed in various tissues [12]. As a matricellular protein, Gal-9 forms lattices on the cell surface shown to interact with other immune cells including T cells and NK cells [13]. Gal-9 is a ligand for T-cell immunoglobin mucin-3 (Tim-3) and it is well known that Tim-3/Gal-9 pathway involved in type 1 helper T cell (TH1) death by inducing intracellular calcium flux [14], whereas low concentrations of Gal-9 (5–30 nM) activated the surviving T cells, to the extent that these T cells proliferate and shift towards central memory and IFN-γ-producing phenotype [15]. In MTB infection, Tim-3/Gal-9 pathway is associated with enhanced production of interleukin (IL)-1β, playing a crucial role in antimicrobial immunity via modulation of the innate inflammatory networks [16].

Tim-3 emerges on the cell surface of fully differentiated TH1 cells [14] that secrete interleukin 2 (IL-2) and IFN-γ, elicit delayed-type hypersensitivity responses, and induce cell-mediated immunity against intracellular pathogens [14]. Tim-3 also expresses on natural killer (NK) cells and can promote the production of IFN-γ in response to Gal-9 [17]. IFN-γ is known to be an inducer of inflammation, necrosis, and apoptosis [18]. IFN-γ up-regulates the mRNA levels of two pro-apoptotic molecules, tumor necrosis factor-alpha (TNF-α) receptor 1 and caspase-8, causing apoptosis in THP-1 macrophages, and at the same time, IFN-γ activates THP-1 macrophages to generate monocyte chemoattractant protein-1 (MCP-1) [19]. Furthermore, IFN-γ can activate Gal-9 expressing on multipotent mesenchymal stromal cells (MSCs), as a result Gal-9 and activated MSCs contribute to immune regulation on T cells [20] and strongly suppress antigen triggered immunoglobulin release [21]. Interferon-γ release assay (IGRA) such as QuantiFERON-TB (QFT) test and ELISPOT is useful to investigate IFN-γ releasing sensitised T cells. We have reported that high level osteopontin is associated with low ELISPOT response, as osteopontin can promote memory T cells to the inflammatory cavitary compartment leading to lesser frequency of memory T cells in the Peripheral blood mononuclear cells (PBMCs) [11]. Thus, in spite of equalization of cell counts from different individuals in ELISPOT assay, the percentage of memory T cells in PMBCs can also impact the positivity. It has been reported that higher percentage of active T cells and effector memory T cells were found in pleural fluid cells (PFCs) compared to PBMCs [22]. More importantly, significantly higher levels of IFN-γ were produced by PFCs than PBMCs following stimulation with TB antigens ESAT-6 or CFP-10 peptides, and polyfunctoional memory CD4 T cells was found in PFCs, featuring enhanced production of multiple cytokines including IL-2 and TNF-α [23]. In other studies, similar to responsive T cells, the memory-like human natural killer (NK) cells expressing the memory-associated marker CD45RO tend to migrate to tuberculous pleurisy [24,25]. The multifacedted effects of Gal-9 indicate the involvement of Gal-9, Tim-3 and IFN-γ in TB pleurisy.

## 2. Results

### 2.1. Immunological Profiling of Plasma and Pleural Fluid in Extrapulmonary Tuberculosis

**Figure 1:**
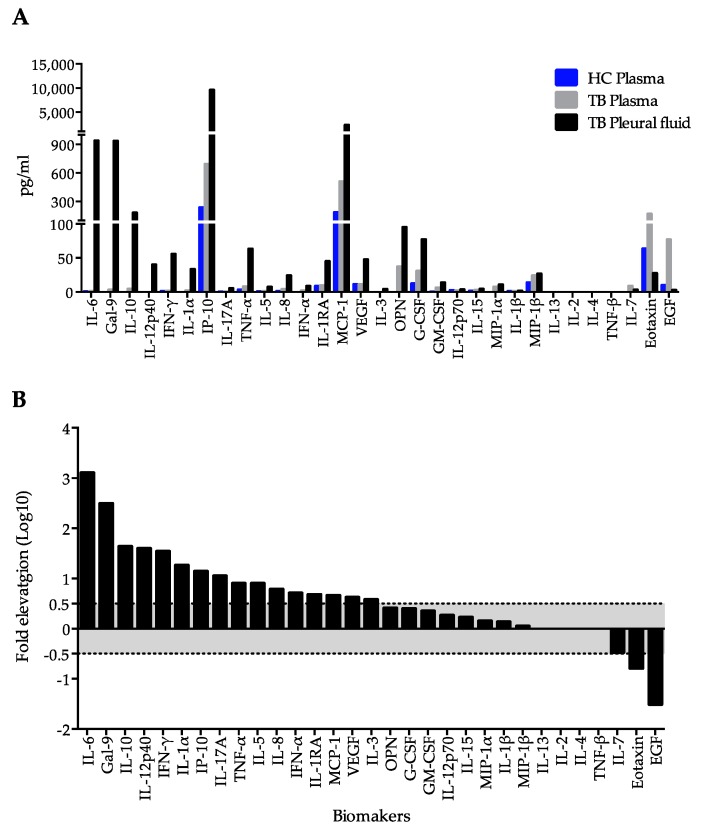
Comparison of cytokine and matricellular protein content between plasma and pleural fluid. (**A**) Values of 31 analytes measured by ELISA or Luminex. Blue panel, a ramdon healthy control, served as a systemic control; (**B**) Panel about the dot line indicate significant decrease or increase of the measured parameters in pleural fluid that were determined as log10 fold change more than 0.5 or less than −0.5, respectively.

Pleural fluid contained significantly higher proportion of lymphocytes compared to plasma and was positive for adenosine deaminase (ADA) (Table 1), whereas the ratios of pleural to plasma lactate dehydrogenase (LDH) and glucose were 0.71 and 0.75, respectively, indicating that lymphocyte-predominant intrathoracic exudate presented in this patient. At the same time, high serum amyloid A in plasma suggested chronic inflammation. Quantitative analysis of cytokines and matricellular proteins (OPN and Gal-9) by Luminex and ELISA, respectively, revealed a remarkable elevation of Gal-9 (936.0 vs. 3 pg/mL), IFN-γ (55.5 vs. 1.6 pg/mL), IL-10 (179.9 vs. 4.2 pg/mL), IL-12p40 (4.0 vs. 0 pg/mL), and VEGF (55.0 vs. 11.3 pg/mL) in patient’s pleural fluid compared to his plasma (Figure 1A). A total of 16 parameters (IL-6, Gal-9, IL-10, IL-12α40, IFN-γ, IL-α, IP-10, IL-17A, TNF-α, IL-5, IL-8, IFNα2, IL-1RA, MCP-1, VEGF and IL-3) were significantly increased in pleural fluid, while three (IL-7, eotaxin and EGF) were decreased (Figure 1B). Although some increase of OPN was observed in pleural exudates compared to plasma (Figure 1A), it was not statistically significant (Figure 1B).

### 2.2. Immunohistochemical Findings

**Figure 2:**
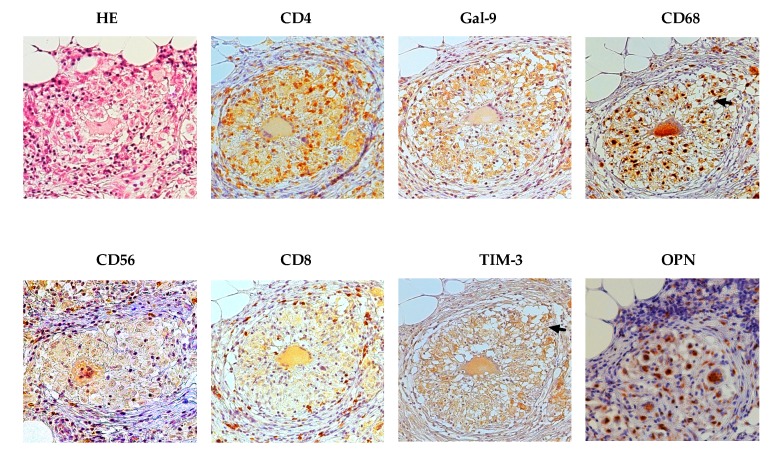
Immunohistochemical staining of peritoneum tissue for CD4, CD8, CD56, OPN, CD68, Gal-9, and TIM-3. HE, hematoxylin and eosin. Magnification, 20×. Arrows indicate loosen tissue at the broken boundary of the granuloma.

Granuloma represents infiltration of inflammatory mononuclear cells in response to MTB infection; while limiting bacterial growth, it also provides a survival niche from which MTB can disseminate. Hematoxylin and eosin (HE) staining of lung tissue extracted from the patient revealed a distinct granuloma with numerous giant cells. Immunohistochemistry analysis showed that the granuloma and surrounding lung tissue were negative for CD20 and CD57 (data not shown), but strongly positive for CD4, Gal-9, CD68, TIM-3, and OPN (Figure 2). Notably, OPN staining was mostly observed in multi-nucleated giant cells surrounded with lymphocytes and epithelioid macrophages. CD8 and CD56 expression was relatively weak and found at the granuloma periphery, whereas CD56 was also stained at the central necrotic core. Unlike the other proteins, TIM-3 was observed both within and outside the granuloma (Figure 2). The TB granuloma is a highly dynamic structure shaped by both host immune factors and the pathogen, and it has been suggested that to secure transmission to a new host, MTB has developed mechanisms to make immune cells destroy the granuloma by apoptosis or necrosis so that its content leaks into bronchial cavities, thus facilitating MTB expectoration and spread [26]. Consistent with this notion, we observed TIM-3 staining in disintegrated ephithelioid macrophages and CD4 positive T cells at the site of granuloma breakage (Figure 2, arrows).

### 2.3. Difference in Interferon-γ (IFN-γ)-Producing Cells between Peripheral Blood Mononuclear Cells (PBMCs) and Pleural Fluid Cells (PFCs)

**Figure 3:**
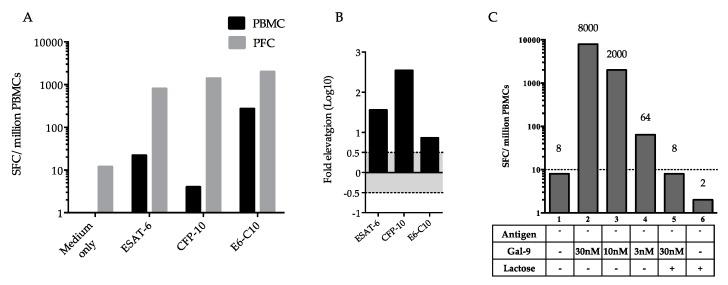
ELISPOT assay of IFN-γ production by PBMCs and PFCs. (**A**) SPCs among peripheral blood mononuclear cells (PBMCs) and pleural fluid cells (PFCs) stimulated with TB antigens; (**B**) Comparison of ELISPOT results between PBMCs and PFCs; black columns show a significant increase in log10 fold elevation of SPCs among PFCs compared to PBMCs; (**C**) Effect of exogenous Gal-9 and lactose in the absence of TB antigens on the number of IFN-γ-producing SPCs. E6-C10, combination of ESAT-6 and CPF-10.

The increase in IFN-γ-producing spot-forming cells (SFCs) in response to ESAT-6, CFP-10, or ESAT-6/CPF-10 was significantly higher among PFCs than among PBMCs (Figure 3A,B). Interestingly, in PFCs not stimulated by TB antigens, exogenous Gal-9 increased, in a dose-dependent manner, the number of SFCs, but this increase was abrogated by lactose, a Gal-9 antagonist (Figure 3C). Out of expectation, lactose alone could even further decrease the background SFCs number in PFCs, indicating that PFCs synthesized Gal-9 that stimulated IFN-γ production (Figure 3C).

### 2.4. Frequency of Gal-9 and Tim3 Positive T Cells in PFCs and PBMCs

**Figure 4:**
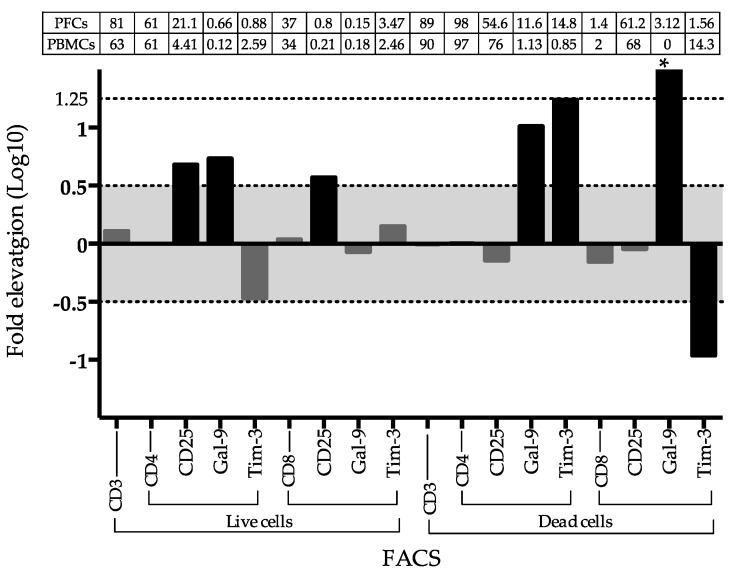
Fluorescence-activated cell sorting (FACS) analysis between PBMCs and PFCs. Fold comparison for the frequency of cells of interest in FACS analysis between PBMCs and FBCs. * indicates infinite as Gal-9 positive expression percentage in dead CD8 T cells from PFCs and PBMCs are 3.12 and 0, respectively. Gates of selection were indicated under x-axis. The absolute percentage of each gated cell group was described in the table above the indicated panel.

Higher frequency of CD25+ cells was observed in both live CD4 and CD8 T cells from PFCs, but only higher frequency of Gal-9+ cells was gated in live CD4+ T PFCs. The frequency of Gal-9 and Tim-3 cells was markedly high in the apoptosis CD4 T+ cells in PFCs compared to PBMCs. The apoptosis CD8+ T cells in PFCs highly express Gal-9, but lacking TIM-3 (Figure 4).

### 2.5. Gal-9 Effects on PFCs Not Stimulated with TB Antigens

**Figure 5:**
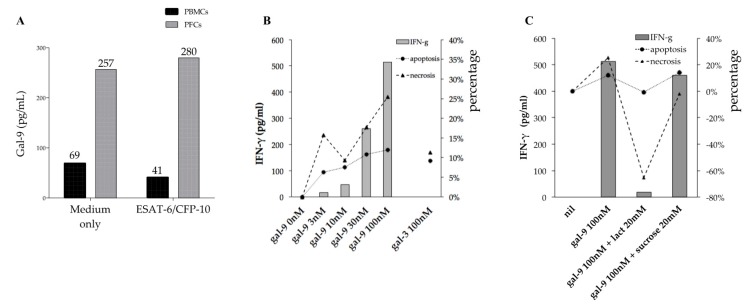
IFN-γ secretion, apoptosis, and necrosis in unstimulated PFC cultures treated with Gal-9. (**A**) Gal-9 levels in supernatants of PBMC and PFC cultures stimulated or not with ESAT-6/CFP-10; (**B**) Dose-dependent effect of Gal-9 on PFC cultures; (**C**) Effect of Gal-9 antagonist lactose on Gal-9 stimulation of PFCs (sucrose was used as control).

The supernatant from the culture of rinsed PFC showed higher levels of Gal-9 compared to that from PBMCs irrespectively of antigen stimulation (Figure 5A). The treatment of PFCs with exogenous Gal-9, but not Gal-3, stimulated IFN-γ production in a linear dose-dependent manner (*y* = 5.1608*x* + 20.28), which corresponded to increased necrosis and apoptosis (Figure 5B). To further investigate the role of Gal-9 in PFCs, they were treated with Gal-9 antagonist lactose for 24 h and analyzed for IFN-γ production, apoptosis, and necrosis. However, no changes were detected (data not shown), indicating a limited effect of endogenous Gal-9 of in vitro culture.

Next, to mimic high Gal-9 levels observed in pleural fluid (Figure 1A), exogenous Gal-9 was added alone or in combination with lactose or sucrose (control for lactose) to PFC cultures. The results indicated that lactose, but not sucrose, is associated with lesser IFN-γ and reduced apoptosis (Figure 5C). Both lactose and sucrose can decrease necrosis, but lactose group showed a markedly increasing cell count compared to the control, as a result −65.4% necrosis was observed (Figure 5C).

### 2.6. Gal-9 Effects on PFCs Stimulated with TB Antigens

**Figure 6:**
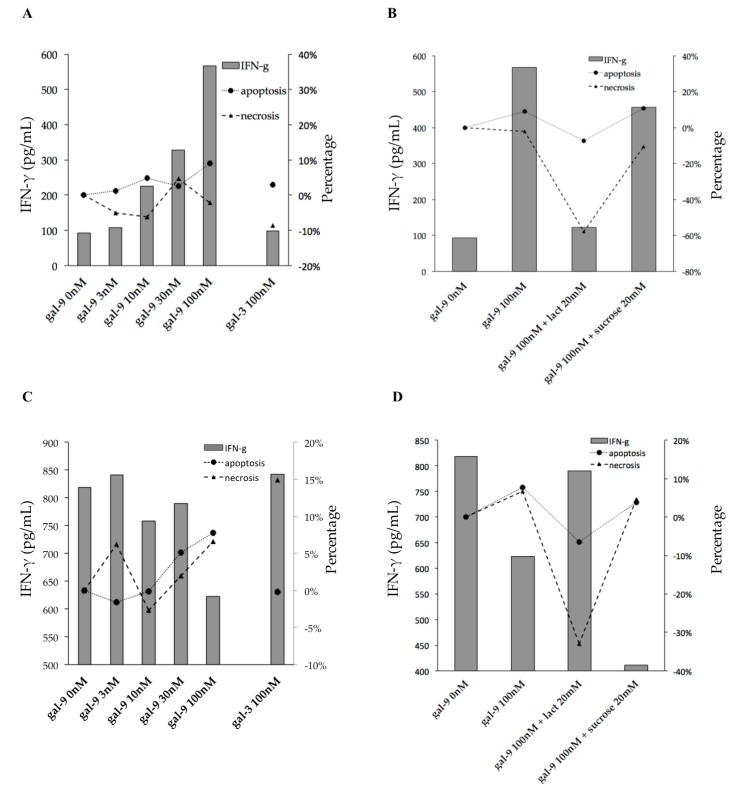
IFN-γ secretion, apoptosis, and necrosis in TB antigen-stimulated PFC cultures treated with Gal-9. (**A**,**B**) ESAT-6/CFP-10 stimulation; (**C**,**D**) PPD stimulation.

In PFC cultures stimulated with ESAT-6/CFP-10, Gal-9 dose-dependently increased IFN-γ secretion and induced apoptosis (Figure 6A), whereas lactose decreased these effects (Figure 6B). However, such activity of Gal-9 was not observed in the PPD-stimulated cultures, except for a significant decrease in IFN-γ secretion at the highest used concentration of Gal-9 (100 mM) (Figure 6C). In contrast to ESAT-6/CFP-10-stimulated cultures, PPD-treated PFCs increased IFN-γ production after the addition of Gal-9 (100 nm) with lactose despite low levels of apoptosis and necrosis (Figure 6D).

## 3. Discussion

To the best of our knowledge, this is the first report of a high Gal-9 level in pleural fluid of a patient with extrapulmonary TB. However, the generality is unclear and was not verified in this study. As Gal-9 involves systemic effusion of a variety of diseases [10,27,28], the mechanism underlying the involvement of Gal-9 in the regulation of pleural effusion is worthy of investigation. In a previous study, Gal-9 staining was observed in TB and sarcoidosis granulomas [29], which is consistent with our results (Figure 2), corresponding to the high frequency of Gal-9+ CD4+ T cells in the pleural fluid (Figure 4) that may have been released from active transmissive granuloma. Tim-3-positive CD4+ T cells, and epithelioid macrophages were found at the region of Gal-9 staining; in addition, enriched TIM-3 expression was detected at the site of granuloma wall disintegration in CD4+ T cells that are likely to result in breakage of the granulomas (Figure 2, arrows) Therefore, these findings suggest activation of Gal-9/TIM-3 signaling induce apoptosis or necrosis of CD4+ T cells (Figure 4) at the point of granuloma breakage, leading to the release of MTB, as well as of matricellular proteins and cytokines, from the granuloma to the adjacent tissue [30].

Moreover, a large number of IFN-γ-producing cells were detected among PFCs, which was also observed in a previous study [23] and which may be related to the increase of cytokines such as IL-6, IL-10 and IL-12 known to affect IFN-γ production [31]. IFN-γ secretion was also induced by PPD stimulation, which, in contrast to ESAT-6/CFP-10 stimulation, was not sensitive to Gal-9 or lactose (Figure 6C,D), indicating involvement of multiple cytokines under PPD stimulation. We became interested in Gal-9 because our previous study suggested the involvement of Gal-9 in the increase of capillary permeability in dengue virus infection [10]. Other galectins (Gal-1 and Gal-3) associated with neoplastic pleural effusion [32,33] may also play a role in enhancing vascular permeability. The upregulation of Gal-9 and TIM-3 expression in T cells was reported in anti-PD-1 therapy-resistant lung cancer patients who had pericardial or pleural effusion [28]. In agreement with the inflammatory environment in the pleural fluid, high level of cytokines (IL-6, Gal-9, IL-10, IL-12α40, IFN-γ, IL-α, IP-10, IL-17A, TNF-α, IL-5, IL-8, IFNα2, IL-1RA, MCP-1, VEGF, and IL-3) were found in the pleural fluid than in the plasma, among which VEGF is of greatest interest, as the involvement of VEGF in the migration of other immune cells to the site [34].

Remarkably, Gal-9 alone was found to enhance the production of IFN-γ in a dose-dependent manner and increase SFCs in ELISPOT. It has been shown that Gal-9 binding to its receptor TIM-3 initiates a signaling cascade leading to apoptosis [35]. IFN-γ induces Gal-9 expression, thus promoting apoptosis [36], while Gal-9 can enhance IFN-γ activity and even synergize with IFN-γ during neuroinflammation [37]. Another study revealed that during mycobacterial infection, IFN-γ acted directly on activated CD4+ T cells, promoting apoptosis [38], which is in agreement with our finding that Gal-9 increased apoptosis and IFN-γ production in PFCs in a dose-dependent manner (Figure 5B and Figure 6A). In addition, immunohistochemistry analysis showed that CD4, Gal-9, and CD68 were expressed in the granuloma (Figure 2), suggesting a role of the TIM-3/Gal-9 pathway in CD4+ T cells and macrophages during pleural effusion.

Since the ELISPOT assay allows focusing on specific sensitized CD4+ T cells [39], it was employed in this study to further dissect the effect of Gal-9 on T cells. It is known that ELISPOT results could be influenced by cytokine release [40], as reported for pro-inflammatory IL-21 which enhanced the response of CD4+ T cells to ESAT-6/CFP-10 in ELISPOT [40]. Therefore, we did not use TB antigen stimulation and found increased IFN-γ production after Gal-9 treatment (Figure 3 and Figure 5). IFN-γ is produced predominantly by CD4+ Th1 effector T cells once antigen-specific immunity develops [41]. Negative results of MTB detection in pleural effusion by culture and PCR in this subject suggest cytotoxic activity against intracellular MTB in pleural fluid [42], which may result from T cell stimulation by Gal-9 in PFCs (Figure 3C) and is consistent with high levels of IFN-γ and Gal-9 present in pleural fluid (Figure 1).

TIM-3 is a surface molecule expressed on immune cells, which serves as a Gal-9 receptor [42]. High frequency of Tim-3+ Gal-9+ cells was found in the apoptotic CD4+ T cells, and Gal-9 alone was found in live CD4+ T cells from PFCs (Figure 2), in support the crucial role of TIM3 in the apoptosis-inducing effect of Tim-3/Gal-9 pathway [42]. On the other hand, the TIM-3/Gal-9 pathway is involved in the activation of human CD4+ T cells and regulation of Th1 and Th17 cytokine secretion. In this process, TIM-3 expression is stimulated only after several rounds of Th1 polarization, and eventually the majority of CD4+ cells become TIM-3-positive [43]. TIM-3 expression does not appear to correlate with cytokine production in already activated CD4+ T cells, but parallels with IFN-γ and IL-17 release by naïve T cells [43]. Therefore, the double-faced effect of Tim-3/Gal-9 pathway may explain the perished T cells at the granuloma site and those proliferating in the pleural fluid. Besides Gal-9, other members of the galectine family, including Gal-1 and Gal-3 can also enhance pleural effusion through immunomodulatory pathways [32].

## 4. Materials and Methods

### 4.1. Patient and Sample Collection

The study was conducted at Tohoku University Hospital and Tohoku University School of Medicine, Sendai, Japan. The study protocol was approved and documented (No. 2010-442, date approval: 2012/10/12) by the Ethics Committee of Tohoku University School of Medicine. All the procedures were conducted in accordance with the Declaration of Helsinki.

**Figure 7:**
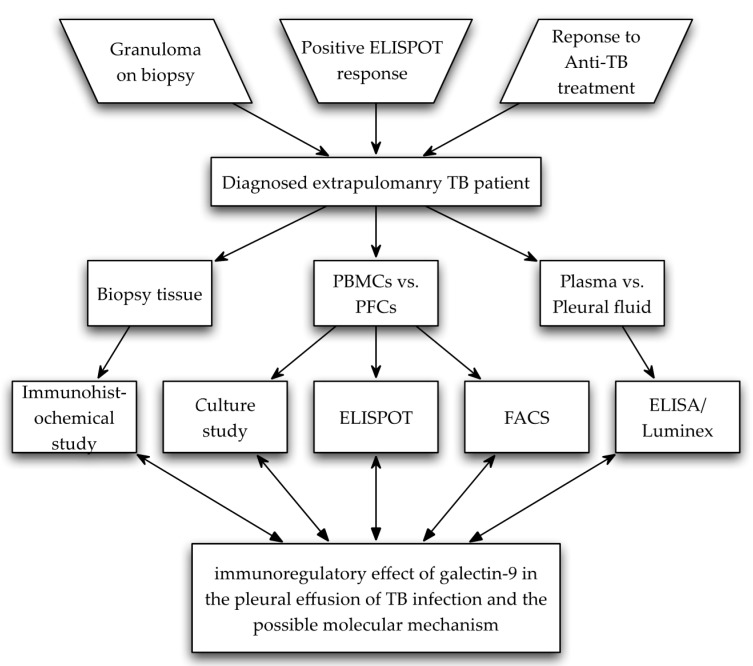
Diagram outline of subject selection, study methods, and purpose of this study.

A 29-year-old extra-pulmonary male TB patient born in Bangladesh was diagnosed according to the WHO guidelines [44]. Typical TB clinical manifestations were observed, including adenosine deaminase (ADA)-positive pleural fluid, despite negative culture result and negative *M. tuberculosis* polymerase chain reaction (PCR) detection of pleural fluid. The patient reacted well to anti-TB treatment later, and fully recovered in the following three years [44] (Figure 7, Diagram). A healthy individual was recruited as control in Luminex and ELISA assay following the standard operating procedure. All participants provided written informed consent for this study.

PFCs were isolated from 120 mL of pleural fluid obtained from the patient. After centrifugation at 3000× *g* for 20 min at 4 °C, the supernatant was analyzed by Luminex or ELISA, and the precipitate was resuspended in 21 mL of Hank’s Buffered Salt Solution (HBSS), and distributed into two 50-mL tubes; then, 10 mL Ficoll-Paque (GE Healthcare Bio-Sciences AB, Uppsala, Sweden) was added into each tube, and the mixture was centrifuged at 400× *g* for 30 min at 20 °C. PFCs were collected at the interface of HBSS and Ficoll-Paque, resuspended in HBSS, washed, centrifuged twice at 600× *g* for 5 min, and then resuspended in AIM V medium (Gibco, Grand Island, NY, USA) (2.5 × 10^5^ per 100 mL) for ELISPOT application, or RPMI medium for in vitro culture or cryopreserved (5 × 10^6^ PFCs/mL/vial). A biopsy sample of peritoneum tissue obtained by exploratory laparoscopy was embedded in paraffin for immunohistochemistry analysis. The peripheral blood was collected from the patient and treated with heparin or EDTA. Same as the procedure for pleural fluid preparation, the EDTA-treated blood (7 mL) was centrifuged within 30 min of collection, and plasma was stored at −80 °C until analysis by enzyme-linked immunosorbent assay (ELISA) or Luminex. PBMCs were isolated from 15 mL of heparinized blood using Ficoll-Paque Plus (GE Healthcare Bio-Sciences AB, Uppsala, Sweden), resuspended in the AIM V medium (Gibco, Grand Island, NY, USA) at the concentration of 2.5 × 10^5^ per 100 mL according to the manufacturer’s recommendation and used for ELISPOT [11] or FACS.

### 4.2. Enzyme-Linked Immunosorbent Assay and Luminex

ELISA and Luminex were performed as previously described [10]. Plasma OPN concentrations were determined using the Human Osteopontin DuoSet ELISA Development System Kit (R&D Systems, Minneapolis, MN, USA). Gal-9 was quantified using the human Gal-9 ELISA kit (Galpharma Co., Ltd., Takamatsu, Japan). A total number of 29 cytokines and chemokines, including epidermal growth factor (EGF), eotaxin, granulocyte macrophage-colony stimulating factor (GM-CSF), G-CSF, IFN-α, IFN-γ, IL-1α, IL-1β, IL-1 receptor antagonist (IL-1RA), IL-2, IL-3, IL-4, IL-5, IL-6, IL-7, IL-8, IL-10, IL-12p40, IL-12p70, IL-13, IL-15, IL-17a, IFN-γ-inducible protein-10 (IP-10), monocyte chemotactic protein 1 (MCP-1), macrophage-inducible protein 1α (MIP-1α), MIP-1β, TNF-α, TNF-β and VEGF, were measured in plasma using the Milliplex Human Cytokine and Chemokine multiplex assay kit (Merck Millipore, Billerica, MA, USA) by the Luminex method. All assays were performed according to manufacturer’s instructions and the results were expressed in pg/mL. Plasma levels of anti-TBGL IgG and IgA were measured using the Determiner TBGL Antibody ELISA kit (Kyowa Medex Co., Ltd., Tokyo, Japan) [45].

### 4.3. Immunohistochemistry

Immunohistochemistry staining was performed as described previously [46] using antibodies specific for OPN (MPIIIB10, DSHB, NIH, Bethesda, MD, USA), Gal-9 (Galpharma Co., Ltd., Japan), Gal-9 receptor TIM-3 (#AF2365, R&D Systems, Minneapolis, MN, USA), macrophage marker CD68 (#PG-M1, DAKO, Tokyo, Japan), B cells marker CD20 (#B-Ly1, DAKO, Tokyo, Japan), NK cell marker CD56 (#123C3, DAKO, Tokyo, Japan), CD57 (TB01, DAKO, Tokyo, Japan), CD4 (#4B12, Nichirei Corp., Tokyo, Japan), and CD8 (#C8/144B, DAKO, Tokyo, Japan).

### 4.4. Enzyme-Linked ImmunoSpot

IFN-γ secreted by stimulated T cells was determined using the Human IFN-gamma ELISPOT Kit (Accession # EL285, R&D, Minneapolis, MN, USA), according to the manufacturer’s instructions. T cells were stimulated with TB antigens, including peptide cassette combination based on the sequence of ESAT-6 or CFP-10. The peptides cassette consisted of 15–20-amino acid peptides designed to cover the whole amino acid sequence of the corresponding antigen; each peptide contained 10 overlapping amino acids (Table 2). To provide water solubility of the peptides, an amino acid tail EEEKKK-OH was added. The peptides were diluted in water and mixed thoroughly before treating cells at the final concentration of 10 µg/mL. To verify the performance of ELISPOT with the synthesized peptides, we compared IFN-γ responses to commercially available ESAT-6/CFP-10 ELISPOT T-SPOT.TB (Oxford Immunotec, Oxford, UK) with those to our ESAT-6 or CFP-10 on 22 PMBC samples from active TB patients [11] (Figure S1). Test results were considered reliable if the numbers of spot-forming cells (SFCs) in positive- and negative-control wells were >20 and <10, respectively. Results were scored as positive if the SFCs number of either ESAT-6 or CFP-10 well was >6. If the total number of SFCs of ESAT-6 and CFP-10 was ≤8, the test result was considered indeterminate. SFCs were counted with an automated Immunospot Analyzer, CTL (Cellular Technologies, Cleveland, OH, USA).

### 4.5. Fluorescence-Activated Cell Sorting 

PBMCs or PFCs cells were first grown in FBS free RPMI medium for 24 h at 37 °C with 5% CO_2_. After 30 min incubation at 4 °C, cells were harvested and stained with PE Annexin V Apoptosis Detection Kit (BD Biosciences), antibodies against human CD3 labeled with PerCP (6 µg/mL, Biolegend, San diego, CA, USA), human CD4 or CD8 labeled with PB (final concentration 6 µg/mL, Abcam, Cambridge, UK), CD25 labeled with PE-Cy (6 µg/mL, BD Biosciences. Inc., Franklin Lakes, NJ, USA), Gal-9 labeled with FITC (1.5 µg/mL, Miltenyi Biotec, Tokyo, Japan), and anti-Tim-3 labeled with APC (1.5 µg/mL, Miltenyi Biotec, Tokyo, Japan). The stained cells were analyzed using a Fluorescence-activated cell sorting (FACS) Calibur Cytometer (BD Biosciences) [47].

### 4.6. Effect of Gal-9 on IFN-γ Production

A volume of 200 µL resupension of PBMCs or PFCs was adjusted to a concentration of 1 × 10^6^/mL in RPMI and stimulated or not with 10 µg/mL ESAT6/CFP10 or purified protein derivative (PPD) (In vitro PPD antigen, BCG Company, Kiyose, Japan) for 24 h in the presence of recombinant human Gal-9 (GalPharma, Takamatsu, Japan) or Gal-3 (#Q6IBA7, R&D, Minneapolis, MN, USA), lactose (128-00095, Wako Pure Chemical Industries Ltd., Osaka, Japan), and sucrose (196-00015, Wako, Japan), and analyzed for Gal-9 (Galpharma Co., Ltd., Takamatsu, Japan) and IFN-γ (R&D Systems, Minneapolis, MN, USA) release to the supernatant by ELISA.

### 4.7. Necrosis and Apoptosis

Necrosis and apoptosis were assessed using Cell Death Detection ELISAPLUS (Roche, Basel, Switzerland), which quantified histone-complexed DNA fragments (mono- and oligo-nucleosomes) in the cytoplasm of apoptotic cells or released from necrotic cells as described [48]. The percentage of apoptotic/necrotic cells was calculated as optical density (OD) (405 nm) in stimulation culture—OD (405 nm) in control/OD (405 nm) in control.

### 4.8. Data Analyses

Statistical analyses were performed using the GraphPad Prism 6.0 software (GraphPad, San Diego, CA, USA). To compare Luminex and ELISA results between plasma and pleural fluid, and ELISPOT measurements of SFC numbers and FACs between PBMCs and PFCs, log10-transformed fold change was used. A significant decrease or increase of the measured parameters in pleural fluid or PFCs were arbitrarily determined as log10 fold change more than 0.5 or less than −0.5, respectively. Graphs were drawn using Microsoft Excel and GraphPad Prism 6.0 software.

## 5. Conclusions

This is the first study to show high levels of Gal-9 in TB pleurisy and the presence of activated IFN-γ-producing T cells in pleural effusion despite the absence of MTB. We also found that Gal-9 alone could increase the number of IFN-γ-producing cells and induce IFN-γ secretion by cultured PFCs. High expression of TIM-3, a Gal-9 receptor, confirms the critical role of Gal-9 in the regulation of IFN-γ production and possibly cell death in pleural fluid, indicating versatile activities of Gal-9 in TB pleurisy. However, to confirm the universality and unambiguity, larger samples including other infectious diseases and granuloma-possessing diseases should be included in future studies.

## Figures and Tables

**Table 1:** Immune characteristics of pleural fluid and plasma.

Parameters	Pleural Fluid	Plasma
OPN (ng/mL)	95	37
Gal-9 (pg/mL)	936	3
IFN-γ (pg/mL)	55.5	1.6
IL-10 (pg/mL)	179.9	4.2
IL-12 (pg/mL)	4.0	0
VEGF (pg/mL)	55.0	11.3
CRP (mg/dL)	n.a	4.3
Amylase (IU/L)	48	70
SAA (μg/mL)	n.a	18.7
Total protein (mg/dL)	5.5	7.7
Glucose (mg/dL)	83	110
LDH (U/L)	153	217
ADA (U/L)	46.9	n.a.
Cell count per μL	2450	n.a.
Lymphocytes (%)	85.7	18
Anti-TBGL-IgG (U/mL)	6.1	3.9
Anti-TBGL-IgA (U/mL)	0.4	0.3

CRP, C-reactive protein; SAA, serum amyloid A (reference 0–10 μg/mL); LDH, lactate dehydrogenase; ADA, adenosine deaminase; TBGL, tuberculous glycolipid antigen; n.a, not applicable.

**Table 2:** Sequence of the peptide cassette.

Antigen	Label	Sequence
ESAT-6	1–20	H-MTEQQWNFAGIEAAASAIQG-EEEKKK-OH
	11–30	H-IEAAASAIQGNVTSIHSLLD-EEEKKK-OH
	21–40	H-NVTSIHSLLDEGKQSLTKLA-EEEKKK-OH
	31–50	H-EGKQSLTKLAAAWGGSGSEA-EEEKKK-OH
	41–60	H-AAWGGSGSEAYQGVQQKWDA-EEEKKK-OH
	51–70	H-YQGVQQKWDATATELNNALQ-EEEKKK-OH
	61–80	H-TATELNNALQNLARTISEAG-EEEKKK-OH
	71–90	H-NLARTISEAGQAMASTEGNV-EEEKKK-OH
	76–95	H-ISEAGQAMASTEGNVTGMFA-EEEKKK-OH
CFP-10	1–20	H-MAEMKTDAATLAQEAGNFER-EEEKKK-OH
	11–30	H-LAQEAGNFERISGDLKTQID-EEEKKK-OH
	21–40	H-ISGDLKTQIDQVESTAGSLQ-EEEKKK-OH
	31–50	H-QVESTAGSLQGQWRGAAGTA-EEEKKK-OH
	41–60	H-GQWRGAAGTAAQAAVVRFQE-EEEKKK-OH
	51–70	H-AQAAVVRFQEAANKQKQELD-EEEKKK-OH
	61–80	H-AANKQKQELDEISTNIRQAG-EEEKKK-OH
	71–90	H-EISTNIRQAGVQYSRADEEQ-EEEKKK-OH
	81–100	H-VQYSRADEEQQQALSSQMGF-EEEKKK-OH

## Supplementary Materials

Supplementary materials can be found at www.mdpi.com/1422-0067/18/7/1382/s1.

## Appendix A

Peripheral blood mononuclear cells (PBMCs) from 22 diagnosed pulmonary tuberculosis patients were tested by using a commercial available kit T-SPOT.TB (Oxford Immunotec, Oxford, UK). The detailed methods for processing these samples are accessible at http://www.mdpi.com/1422-0067/18/1/19/htm. The PBMCs from same 22 subjects were also tested by using the homebrew peptides and IFN-γ release assays, and then compared the results to those obtained using commercial available kit, by linear regression. A significant regression was assumed at *p* < 0.05 (Figure S1A,B). A graphical method Bland-Altman plot was applied to compare these two measurements, which was achieved by using MedCalc statistical software Version 16.8.4 (Ostend, Belgium). Only one spot out of 22 in ESAT-6 or CFP-10 measurement exceeded the maximum allowed difference, and the *p* value of each is less than 0.05, indicating a significant agreement between commercial and homebrew method for detecting ESAT-6 or CFP-10 specific reacting spots (Figure S1C,D).
