# Supplementary material for: Secretion of IFN-γ Associated with Galectin-9 Production by Pleural Fluid Cells from a Patient with Extrapulmonary Tuberculosis
Source: Int J Mol Sci. 2017 Jun 28;18(7):1382. doi: 10.3390/ijms18071382 (PMC5535875; doi:10.3390/ijms18071382)
Supplement: Supplementary file 1 [file ijms-18-01382-s001.pdf]

Secretion of IFN-γ Associated with Galectin-9 Production by Pleural Fluid Cells from a Patient with Extrapulmonary Tuberculosis

Jingge Zhao, Beata Shiratori, Haorile Chagan-Yasutan, Makoto Matsumoto, Toshiro Niki, Michinori Tanaka, Yayoi Takahashi, Osumu Usami, Yugo Ashino, and Toshio Hattori

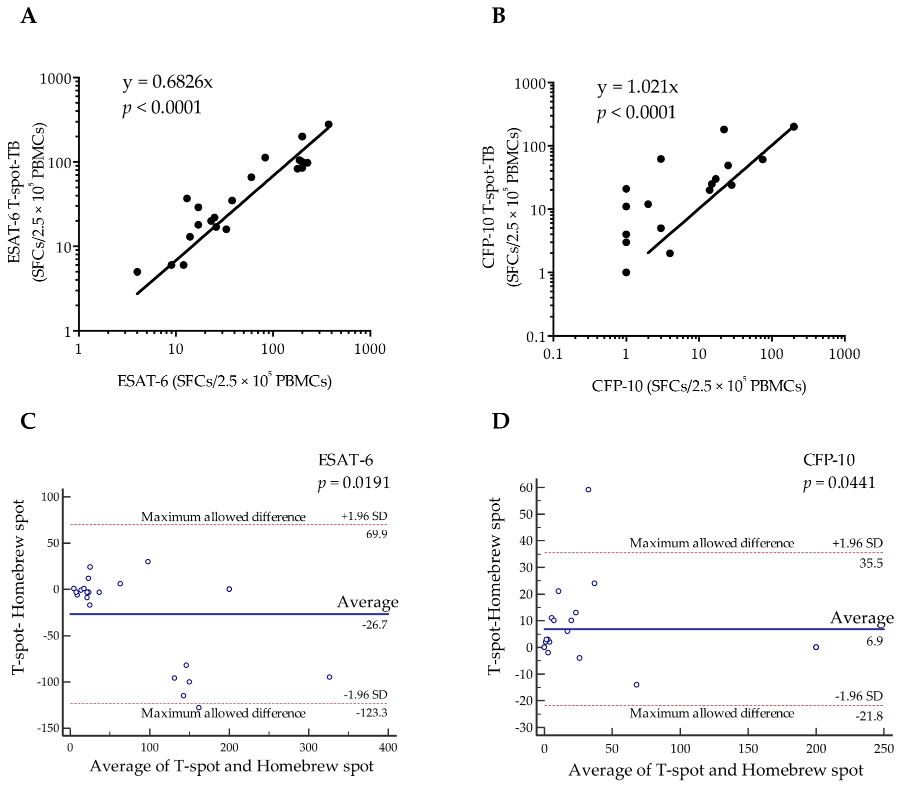

Figure S1. Comparison of ESAT-6 and CFP-10 peptides used in the study with commercially available antigens. (**A, B**) Linear regression between homebrew antigens-based and commercial T-spot antigen-based assay; (**A**) ESAT-6, (**B**) CFP-10. (**C, D**) The agreement between homebrew antigens-based and commercial T-spot antigen-based assay were analyzed by Bland-Altman plot method; (**C**) ESAT-6, (**D**) CFP-10.
